# Diminutive Ureteral Stone Causing Caylyceal Rupture: Case Report and a Review of the Treatment Options

**DOI:** 10.7759/cureus.39644

**Published:** 2023-05-29

**Authors:** Abdallah Khashan, Sadat Kasanga, ZakaUl Haq, Gagandeep Saini, Samer Talib, Sumayah Derbala, Michael Carson

**Affiliations:** 1 Internal Medicine, Raritan Bay Medical Center, Perth Amboy, USA; 2 Biology, Liberty University, Centreville, USA

**Keywords:** nephrolithiasis, calyceal rupture, urolithiasis, urinoma, kidney calculi

## Abstract

Rupture of the renal collecting system is a rare event, usually occurring at the ureterovesical junction (UVJ). The most common cause is nephrolithiasis, usually directly correlated with the size of the stone. Other causes include bladder outlet obstruction, ureteropelvic junction obstruction, and extrinsic ureteral compression by a malignant pathology. The mechanism is increased pressure within the collecting system, and symptoms vary from vague mild abdominal pain to severe excruciating pain. We present a case of a 19-year-old female with obstructive uropathy and renal calyceal rupture caused by a 3 mm stone at the UVJ. Due to the small size of the stone and her hemodynamic stability, she was treated conservatively with tamsulosin and IV ceftriaxone. The following day she passed sediment in the urine and noted pain improvement. Calyceal rupture with small stones is exceedingly rare, may be missed on a CT without contrast, and should be suspected when perinephric edema or fluid is seen. This is the smallest recorded stone causing calyceal rupture to the best of our knowledge. CT with contrast is indicated for diagnosis when calyceal rupture is suspected and is suggested by extravasation of contrast. Early diagnosis and intervention, in collaboration with urologists, can help to avoid long-term complications such as acute kidney injury, urosepsis, and urinoma. Conservative management may still be considered after a calyceal rupture in patients with small, potentially passable stones. However, if there is associated obstructive uropathy, infection, or significant rupture, then stenting may be indicated. This case highlights the diagnosis of calyceal rupture in the setting of tiny stones and the efficacy of conservative therapy versus early stenting in the management of stable patients.

## Introduction

Renal colic is a common cause of presentation to the emergency department, with an estimated prevalence of 10% to 15% of the United States population and resulting in over 1.3 million visits to the emergency department annually [1]. It typically presents with severe left or right flank pain that typically originates over the costovertebral angles and radiates toward the groin. It is most often caused by kidney stones, which are present in 1.7-14.8% of the population [2]. Risk factors for kidney stones include volume depletion, hypercalciuria, hyperoxaluria, hyperuricosuria, and hypocitraturia [3].

Acute renal colic management has three components: pain management, treating the cause, and managing complications. CT scan without contrast is the modality of choice to diagnose kidney stones; however, this case illustrates a rare complication of a small stone where a contrast study is indicated to clarify the diagnosis of calyceal rupture, 74% of which are related to obstructing stones [4]. While invasive stenting was not required in this case, we will review the diagnosis and management of calyceal rupture to improve internists' ability to recognize, diagnose, and manage this infrequent event collaboratively with the urology team.

## Case presentation

**Figure 1:**
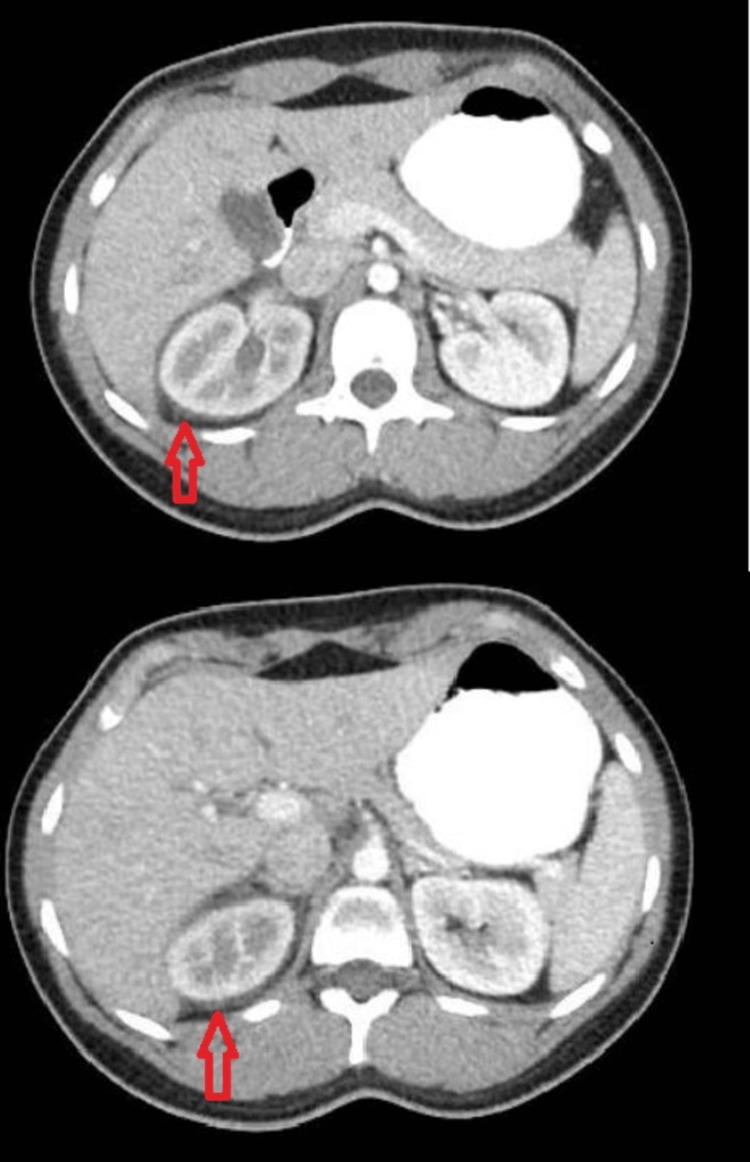
CT abdomen and pelvis with contrast shows perinephric edema/ fluid collection (arrowhead) around the right kidney

**Figure 2:**
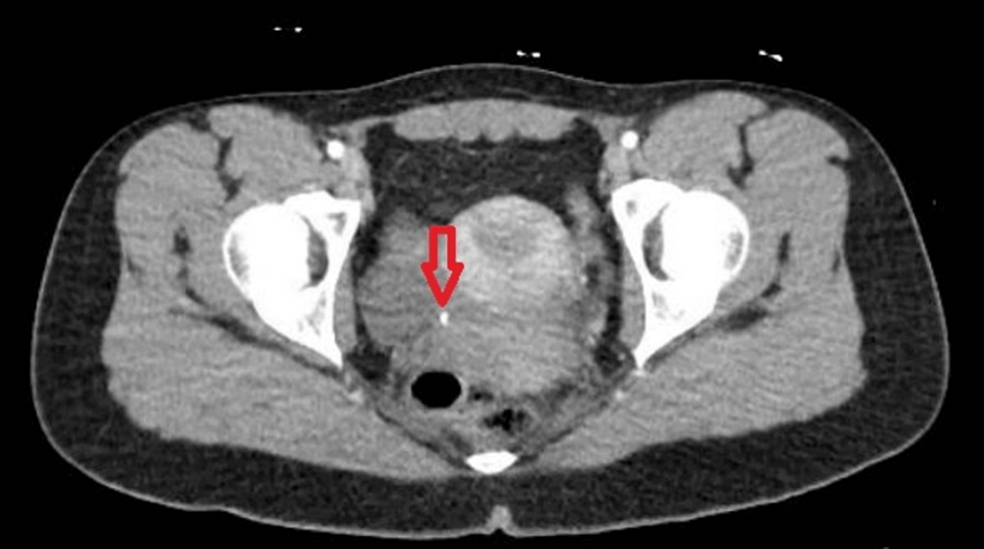
CT abdomen and pelvis showing 3 mm calculus at the ureterovesical junction

A 19-year-old woman with no past medical issues presented with acute right-sided lower abdominal pain radiating to the groin with associated nausea and vomiting. She endorsed chills but no fever, hematuria, dysuria, nausea, or vomiting. Initial vital signs in the emergency department: blood pressure 106/57 mmHg, heart rate 82 beats/minute, oxygen saturation 99% on room air, temperature 98.3 F, and respiratory rate 18/minute. Physical examination was notable for right costovertebral angle tenderness. Urinalysis showed white blood count (WBC) 30-50/HPF, RBC 3-5/HPF, blood - large, bacteria - rare, leukocyte esterase - small, and nitrite was negative. A pregnancy test was negative, serum WBC 13,200 m/mm^3 ^with 71.4% neutrophils. Computed tomography of the abdomen and pelvis with contrast showed a 3 mm calculus at the ureterovesical junction with mild to moderate right hydroureteronephrosis and edema adjacent to the right kidney (Figures 1, 2), indicating a ruptured calyx.

She was treated conservatively with IV fluids, intravenous ketorolac as needed, IV ceftriaxone, tamsulosin, and a urology consult was placed. The urology consultant decided to treat her conservatively as she was afebrile, hemodynamically stable, and did not meet the criteria for sepsis. Additionally, given the size of the stone being 3 mm, there was a good chance it would pass spontaneously, and she could avoid the potential risks of an invasive procedure. If she developed new symptoms, signs of infection, or the stone did not pass the next day, the consultant's initial plan was to place a ureteral stent. The patient was closely monitored, and after 36 hours, she reported passing "sediment" in her urine and experiencing significant improvement in the pain. The following day she denied any urinary symptoms, was discharged on tamsulosin, and was advised to follow up with the primary care physician and urologist as an outpatient.

## Discussion

Renal colic usually manifests by acute unilateral abdominal pain usually associated with hematuria, nausea, and vomiting. Treatment focuses on symptomatic treatment, expulsive therapy for the stones, and managing complications, including superinfection, hydronephrosis, and rarely calyceal rupture.

Pain management options include acetaminophen, IV nonsteroidal anti-inflammatory drugs (NSAIDs), or narcotics. IV NSAID is the first choice in the case of normal renal function [5] because they were associated with fewer vomiting episodes and reduced incidence of rescue IV NSAID administrations in comparison with acetaminophen and morphine [5].

Small stones ­of <5 mm typically pass spontaneously and so are treated conservatively with pain medications, smooth muscle relaxants like tamsulosin, and IV antibiotics if a superimposed infection is suspected.

Only 60% of stones 5-7 mm pass spontaneously without any intervention [6], and the passage rates for stones sized 7-9 mm and >9 mm are 48% and 25%, respectively [6]. Expulsive therapy is advised in stones less than 10 mm as it is more cost-effective and increases the rate of stone passage without intervention [7]. Multiple agents can be chosen, including alpha-blockers, calcium channel blockers, and 5-phosphodiesterase inhibitors. Tamsulosin is the most effective alpha-blocker in aspects of expulsion rate, expulsion time, and safety [7]. Renal stones >10 mm require urology consultation and may require interventions like shockwave or direct lithotripsy and percutaneous nephrolithotomy, while large ureteral stones require ureteroscopy or shockwave lithotripsy. Ureteroscopy is associated with a higher stone passage rate, especially in distal stones, although it is associated with a higher risk of complication [8].

Renal calyceal rupture is a rare manifestation of stones <5 mm, as 98% of them pass spontaneously without causing obstructive uropathy [9]. In the two percent of cases where stones <5 mm do cause obstruction, typically, it is not associated with calyceal rupture. This case is unique in that a stone of 3 mm caused both obstructive uropathy and calyceal rupture. To our knowledge, this is one of the smallest stones reported to cause calyceal rupture [10].

The best diagnostic study for the initial identification of kidney stones is a CT scan without contrast [11], especially in cases with acute kidney injury. However, it may be difficult to visualize calyceal rupture without contrast. The clinical presentation of forniceal rupture varies greatly and may be indistinguishable from renal colic [12]. The presence of perinephric edema or fluid collection (Figure 1) on initial imaging suggests the presence of calyceal rupture and should raise suspicion for further imaging. This can be confirmed by a CT scan with contrast to detect contrast leakage and/or urinoma formation. Contrast-enhanced CT with delayed phase protocol has a higher sensitivity as it shows any asymmetric delayed contrast secretion at the site of obstruction. It also helps to differentiate between small kidney stones and vascular calcifications [13]. Regardless of the stone size, a urology consult is advised to evaluate calyceal rupture regarding the need for surgical management. Conservative treatment can be considered in uncomplicated cases [14]. Early urological evaluation for possible invasive intervention should be considered by the internist team, especially with stones >5 mm.

Indications for interventions are presenting with severe agonizing pain not adequately controlled by analgesics, increased fluid/edema around the kidney in comparison with the initial study, complicated urinoma, an acute rise in creatinine level, or superinfection [14]. Interventions may include double-J ureteral stents, ureteroscopy, percutaneous nephrostomy catheters, or urinoma drainage, depending on the site of obstruction [14].

## Conclusions

Calyceal rupture is a rare but recognized complication of small kidney stones. CT with contrast is advised if perinephric edema is noted in the initial imaging study. A urology consult should be placed for possible intervention in the setting of acute kidney injury, secondary infection, urinoma formation, inadequate pain control, or increasing size of the fluid collection around the kidney. This case reviewed the diagnosis of calyceal rupture, an infrequent occurrence with small stones, and when to consider conservative management versus urgent urological consultation in order to avoid a decline in patient status.
